# 外泌体在肺癌发病及诊断中作用的研究进展

**DOI:** 10.3779/j.issn.1009-3419.2020.104.18

**Published:** 2020-07-20

**Authors:** 焕焕 毕, 敦强 任, 君 张, 红梅 王

**Affiliations:** 266000 青岛，青岛大学附属医院呼吸与危重症医学科 Department of Respiratory and Critical Care Medcine, The Affiliated Hospital of Qingdao University, Qingdao 266000, China

**Keywords:** 肺肿瘤, 外泌体, 诊断, 预后, 生物标志物, Lung neoplasms, Exosomes, Diagnosis, Prognosis, Biomarker

## Abstract

世界范围内肺癌的发病率居高不下，不论男性还是女性，肺癌均是恶性肿瘤死亡的首位原因。肺癌的早期诊断将显著改善患者的预后，寻找辅助早期肺癌诊断的标志物是当前研究热点。外泌体是由各种细胞分泌的纳米级微囊泡，包含核酸、蛋白质和脂质等多种成分，是细胞间或细胞与组织间信号传递或物质运输的重要载体。独特的富集机制使其具有作为生物标志物的稳定性和特异性。外泌体除参与肺癌肿瘤微环境的形成及新生血管的生成外，还参与化疗、靶向治疗反应及预后评估。诸多研究进展成果为延长肺癌患者生存期带来新的希望。本文就外泌体特异性蛋白和微小RNA（microRNA, miRNA）在肺癌发病、诊断及预后评估中的价值进行综述。

## Abstract (en)

The incidence of lung cancer is high worldwide, and lung cancer is the leading cause of death from malignant tumors in both men and women. Early diagnosis of lung cancer can significantly improve the patient's prognosis. Therefore, searching for specific markers to assist in the early diagnosis of lung cancer is urgent question. Exosomes are nano-sized microvesicles and contain various biomaterial, including nucleic acids, proteins, and lipids. Exosomes are important carriers of these biomaterial, serve important roles in intracellular communications and signal transduction among tissues. Due to its unique enrichment mechanism, it has the stability and specificity as a biomarker. Exosomes are not only involved in the formation of tumor microenvironment and new blood vessels in lung cancer, but also involved in chemotherapy, targeted therapy response and prognosis assessment. Many research advances bring new hope for prolonging the survival of lung cancer patients. This article reviews the value of exosome specific protein and microRNA (miRNA) in lung cancer in the diagnosis and prognosis of lung cancer.

肺癌是世界范围内导致恶性肿瘤死亡的首位原因^[1]^，肺癌占所有肿瘤的21%，占所有肿瘤死亡的27%。2015年全球统计数据表明，每年约新增加180万例肺癌患者，死亡160万例，其中中国贡献了35.8%的新增病例和37.6%的死亡病例^[2]^。Ⅰ期非小细胞肺癌（non-small cell lung cancer, NSCLC）患者手术后的5年生存率高达83%，但由于缺乏早期特异性诊断标志物^[3]^，大多数肺癌患者确诊时已处于晚期，NSCLC的5年生存率不足5%。近年来肺癌的诊断方法不再局限于组织活检，液体活检已广泛应用于临床，外泌体作为液体活检的一种，它在癌细胞出现再循环之前早有分泌，且其含量及生物分子构成存在变化^[4, 5]^。由于外泌体受囊泡及非囊泡蛋白复合物的保护，其生物成分不易遭到破坏^[6, 7]^，同时它还可通过激活特定的癌基因或灭活抑癌基因来调节受体细胞的活性^[8]^，故具备作为生物标志物的稳定性及特异性。外泌体水平与肺癌患者总生存率呈负相关^[9]^，有研究^[10]^表明外泌体直径比临床分期能更准确地预测肺癌复发可能性，可认为是与预后相关的独立危险因素。基于诊断技术的进步，靶向及免疫治疗的临床应用打破了肺癌传统单一的治疗模式，当前肺癌患者5年生存率已提高至15.5%^[11]^。本文将从外泌体表征在肺癌发生发展、诊断及预后中的作用展开综述。

## 1 外泌体的概述

1984年，Harding等^[12, 13]^在研究网织红细胞分化时首先观察到内吞起源的纳米囊泡的存在，这些小泡参与了网织红细胞表面转铁蛋白受体的去除，研究者因此推测该囊泡在红细胞成熟过程中发挥着潜在作用。这些细胞外囊泡包括外泌体、凋亡小体和微囊泡，其中凋亡小体相对容易分离，而由于外泌体的直径在40 nm-180 nm之间，微囊泡的直径在100 nm-1, 000 nm之间^[14-18]^，二者的直径范围存在交叉，以目前外泌体分离技术很难完全彻底分离，因此国际细胞囊泡协会（International Society for Extracellular Vesicle, ISEV）允许使用外泌体代替细胞外囊泡一词。

## 2 外泌体的表征

### 2.1 外泌体的生物起源

外泌体由不同细胞释放，包括所有真核生物（如阿米巴变形虫、秀丽隐杆线虫、哺乳动物等）及真核细胞（免疫细胞、干细胞、肿瘤细胞等）；外泌体也可从多种体液中分离出来，包括血浆^[19]^、尿液、支气管肺泡灌洗液（bronchoalveolar lavage fluid, BALF）^[20]^、唾液、母乳、羊水、腹水、脑脊液、胆汁和精液等。外泌体通过内吞作用形成早期内吞小体，然后以内生出芽的方式形成多个小囊泡，并选择性地接受胞浆内的蛋白质和脂质形成晚期内吞小体，即多泡体（multivesicular bodies, MVB）。多泡体在转运必需内吞体分选复合物（endosomal sorting complex required for transport, ESCRT）和相关蛋白的调控下^[21]^，一部分MVBs与溶酶体融合后降解，另一部分与质膜融合后以外泌体的形式释放到细胞外^[22]^。

### 2.2 外泌体的组成与功能

有报道^[23]^已证实，外泌体的主要成分是蛋白质和核酸。蛋白质可分为参与其结构形成的结构蛋白及作为外泌体标记的特异蛋白，如CD9、CD63、CD81和CD82等^[24]^。核酸包括微小RNA（microRNA, miRNA）、DNA、信使RNA（messenger RNA, mRNA）、长链非编码RNA（long noncoding RNA, lncRNA）等，研究^[25]^表明外泌体核酸可被转运到邻近或远处受体细胞产生生理或病理效应，如介导细胞信息交流、信号转导、运输遗传物质和调节免疫反应等。外泌体参与肺癌发生发展、侵袭及预后的各个环节。

## 3 外泌体蛋白在肺癌发生、发展、诊断及预后中的作用

### 3.1 外泌体蛋白在肺癌发生发展中的作用

外泌体蛋白参与肺癌发生发展过程，包括缺氧微环境的形成、新生血管的形成及躲避宿主免疫细胞的攻击。缺氧是肿瘤微环境的标志，缺氧本身会诱导炎性分子的增加，如白介素（interleukin, IL）-6、IL-1、肿瘤坏死因子α（tumor necrosis factor alpha, TNF-α）和IL-10等^[26]^，另有外泌体热休克蛋白-70（heat shock protein 70, HSP70）激活核转录因子-kappa B（nuclear factor-kappa B, NF-κB）信号后，间充质干细胞分泌大量的趋化因子CCL（chemokine C-C motif ligand）-2、CCL-7、CCL-12，增加微环境中的炎性因子浓度，招募单核细胞、巨噬细胞在肿瘤部位聚集，塑造肿瘤免疫抑制环境^[27, 28]^。新生血管是肿瘤发生发展的关键，该过程贯穿肿瘤侵袭及转移始终。缺氧条件下肿瘤外泌体增加的同时诱导血管形成^[29]^，肿瘤来源的外泌体本身含有的转化生长因子（transforming growth factor, TGF）及激活巨噬细胞分泌的血管内皮生长因子（vascular endothelial growth factor, VEGF）促进血管生成^[18]^。上皮-间质转化（epithelial-mesenchymal transition, EMT）是肿瘤进展过程中血管生成及转移形成的关键因素。EMT的主要特征是E-钙黏蛋白表达减少，此蛋白上调后A549肺腺癌细胞的生长速度有所减缓，反之则A549癌细胞的迁移和侵袭增强^[30]^。有研究^[31]^显示，含Rab3蛋白的外泌体过表达将会激活细胞内蛋白激酶B（protein kinase B, PKB/Akt）和糖原合成酶激酶3β（glycose synthase kinase 3 beta, GSK3β）信号通路降低E-钙黏蛋白的水平，诱导EMT过程加快肿瘤进展。癌相关成纤维细胞（cancer-associated fibroblasts, CAFs）可通过外泌体将SNAIL重组蛋白1传递给受体癌细胞，诱导肺癌细胞发生EMT，促进肿瘤血管生成^[32]^。YKT6是参与外泌体产生与释放的一种SNARE蛋白，阻断YKT6后外泌体释放减少及肿瘤转移被抑制^[33]^。肿瘤进展的另一个关键步骤是逃避宿主免疫系统监视，研究发现NSCLC血浆外泌体程序性死亡因子配体（exosomal programmed cell death ligand 1, ePD-L1）作用于T细胞表面的程序性死亡因子（programmed cell death-1, PD-1）抑制T细胞的活化^[34]^，同时扩增的调节性T细胞通过细胞外结构域使细胞毒性T细胞（cytotoxic T-cell lymphocytes, CTLs）失活，造成CTLs免疫抑制及延长癌细胞生存，诱导癌细胞的免疫逃逸^[35, 36]^。Poggio可通过阻断外泌体PD-L1与PD-1的结合来提高CTLs活性、抑制肿瘤细胞的免疫逃逸及增强抗肿瘤作用^[37]^。T细胞免疫球蛋白-3（T cell immunoglobulin-3, Tim-3）是新一代的免疫检查点，被其配体Galectin-9激活后可负性调节CD8/NK细胞的免疫应答^[38]^，NSCLC患者血浆外泌体Tim-3及配体明显升高，且与肿瘤体积增大、进展、淋巴结及远处转移呈正相关^[39]^。

### 3.2 外泌体蛋白在肺癌诊断中的作用

外泌体特异性蛋白可作为肺癌诊断的生物标志物，也可反映肺癌治疗效果。有研究^[40]^对晚期NSCLC患者和正常人血浆外泌体蛋白进行分析，结果表明外泌体特异性蛋白CD91在前者中显著增加，其检出能力也高于现有的生物标志物癌胚抗原（carcino-embryonic antigen, CEA），且对于外泌体的标记性蛋白而言，CD9、CD63和CD81联合检测更具有特异性^[41]^。研究^[42, 43]^表明NSCLC患者血浆外泌体α-2-HS-糖蛋白（alpha2-HS-glycoprotein, AHSG）、细胞外基质蛋白1（extracellular matrix 1, ECM1）、CD151、TSPAN8（tetraspanin 8）和CD171含量高于健康对照组；血清外泌体磷脂酰肌醇蛋白聚糖1（glypican 1, GPC1）、CD91、CD317在肺癌组的表达水平高于健康对照组^[40, 44]^。MUC1是由MUC1基因表达的一种高糖基化蛋白，外泌体MUC1可区分NSCLC与健康者，同时该研究还发现血浆MUC1的表达水平与健康人无差异^[45]^，Yamashita等^[46]^研究出现类似结果，血浆外泌体表皮生长因子受体（epidermal growth factor receptor, EGFR）水平明显高于对照组，血浆EGFR水平与对照组未见明显差异。肺癌的免疫指标与外泌体相关，肿瘤细胞ePD-L1一定程度上可反映肿瘤组织程序性死亡因子配体1（programmed cell death ligand 1, PD-L1）的表达水平^[47]^，见表 1^[40-47]^。外泌体在肺癌患者预后评估中也有重要价值。外泌体蛋白EGFR阳性的患者对表皮生长因子受体酪氨酸激酶抑制剂（EGFR tyrosine kinases inhibitor, EGFR-TKI）药物治疗有较高的客观应答率和较长的无进展生存期^[48, 49]^，EGFR和P53突变者外泌体含量增加，外泌体EGFR浓度与总生存期呈负相关，浓度升高生存期缩短^[50]^。另有研究表明外泌体NY-ESO-1、EGFR、胎盘碱性磷酸酶（placental alkaline phosphatase, PLAP）、上皮细胞黏附分子（epithelial cell adhesion molecule, EpCAM）、Alix和脂多糖结合蛋白（lipopolysaccharide binding proteins, LBP）与生存期及预后呈负相关，其中血浆NY-ESO-1是多次实验后被证明与总生存期显著相关的特异性蛋白^[51, 52]^，血清外泌体LBP上调提示早期NSCLC患者发生转移的可能性大，反之亦然^[52]^。

**1:** 外泌体特异性蛋白质在肺癌中的临床意义 The clinical significance of exosomal pecific proteins in lung cancer

The author, year	Sample type	Sample size	Extract method	The miRNA species	Clinical significance
Ueda K. *et al*, 2014^[40]^	Serum	165 LC *vs* 64 HC *vs* 29 IPD	IMS	CD91, CD317, CD91, Itgα2b	Up-regulation: CD91 and CD317. Distinguish lung cancer, healthy people and interstitial lung disease.
Jakobsen KR, 2015^[41]^	Plasma	109 NSCLC *vs* 9 HC	EV array	37 proteins	Up-regulation. Combined detection of CD9, CD63, and CD81 increased the specificity of NSCLC screening.
Niu L. *et al*, 2018^[42]^	Plasma	125 NSCLC *vs* 46 HC	AUC	AHSG, ECM1, CEA	Up-regulation. Distinguish between NSCLC and healthy people.
Sandfeld-Paulsen B. *et al*, 2016^[43]^	Plasma	431 LC *vs* 150 HC	EV array	10 proteins	Up-regulation: CD151, CD171, TSPAN8. Distinguish between NSCLC and healthy people.
Meng XK. *et al*, 2016^[44]^	Serum	18 NSCLC *vs* 9 HC	AUC	GPC1	Up-regulation. Distinguish between NSCLC and healthy people.
Pan D. *et al*, 2019^[45]^	Plasma	27 LC *vs* 16 HC	AUC	3, 235 proteins	Up-regulation. In the lung cancer group, the increase was 1.5 times.
Yamashita T. *et al*, 2013^[46]^	HPAEpiC, HARA-B cells cultures	NA	IMS	EGFR	Up-regulation. Exosomal EGFR levels were significantly elevated in 5/9 lung cancers.
Peng XX. *et al*, 2019^[47]^	Plasma	H226, H1975 *vs* MCF-7	AUC	PD-L1	When the expression of ePD-L1 is positive, it reflects the expression level of PD-L1 in tumor tissue.
LC: lung cancer; HC: healthy control; IMS: immunomagnetic bead-based separation; NSCLC: non-small cell lung cancer; Itgα2b: integrin alpha-IIb; EV array: extracellular vesicle array; AUC: analytical ultra centrifugation; AHSG: alpha 2-HS-glycoprotein; ECM1: extracellular matrix; CEA: carcino-embryonic antigen; TSPAN8: tetraspanin 8: IPD: interstitial lung disease: GPC1: glypican 1; EGFR: epidermal growth factor receptor; PD-L1: programmed death ligand-1.					

## 4 外泌体miRNA在肺癌发生、发展及诊断中的作用

### 4.1 外泌体miRNA在肺癌发生发展中的作用

miRNA表达异常与肿瘤形成、细胞增殖、侵袭和血管生成有关，miRNAs作为肿瘤抑制因子或者癌基因参与肿瘤的血管生成过程^[53]^。研究表明外泌体通过miRNAs刺激新生血管内皮细胞生长^[54]^，miR-210上调基质细胞中ephrinA3的表达促进血管形成^[55]^，miR-9转入内皮细胞后降低细胞因子信号转导抑制因子（suppressor of cytokine signaling, SOCS）-5的水平，诱导JAK-STAT通路的激活，促进内皮细胞迁移与肿瘤血管生成^[56]^。外泌体miR-222-3p靶向作用于SOCS3的启动子增加肿瘤细胞的增殖及自身抗凋亡能力^[57]^，并非促进新生血管增加的miRNA都呈上调趋势，miNA-373、miNA-512下调促进肿瘤血管生成^[58]^。外泌体在恶性肿瘤中的作用非肺癌所特有，不同研究之间成果可相互借鉴，乳腺癌进展期高表达的miR-660-5p已被证明在NSCLC患者外泌体和血浆中也高表达，且miR-660-5p通过靶向作用于Krüppel样因子4（Krüppel-like factor 4, KLF4）介导肺癌细胞分化、增殖和凋亡过程^[59]^。目前外泌体miRNA相关报道大部分标本为血清或血浆，BALF是一种重要的近端生物体液，相对于肺组织活检而言，持续BALF检查不仅可降低肺癌的播散风险和转移率，同时对距主支气管较远的病变定性也有重大意义^[60]^。因此BALF外泌体miRNAs在肺癌早期诊断中的潜在价值引起了越来越多研究者的重视。吸烟是肺癌发生的重要因素，研究^[61]^证实BALF外泌体特征与吸烟密切相关，吸烟者BALF外泌体miRNAs表达谱发生改变，miRNA表达谱显示吸烟者miR-let-7e、miR-let-7g和miR-26b表达明显减少，但尚不具备作为生物标志物的潜力。

### 4.2 外泌体miRNA在肺癌诊断中的作用

外泌体miRNA可作为肺癌诊断的生物标志物，也可作为反映肺癌治疗效果的生物标志物。并非所有miRNA都具备作为生物标志物的条件，外泌体选择性包裹某些miRNAs并通过其独特的机制增加特定种类miRNAs的丰度^[62-82]^。2006年Yanaihara等^[64]^全基因组测序表明NSCLC患者肺组织中有12种特异性miRNAs，2009年Rabinowits等^[66]^证实NSCLC患者血浆外泌体miRNA谱与之一致。外泌体miRNA在肺癌患者与健康人之间存在差异，肺部不同占位性及不同病理类型之间存在差异，Rodríguez等^[76]^研究表明BALF和血浆外泌体特异性miRNA种类不同，见表 2^[65-82]^。外泌体除了作为肺癌诊断的标志物外，还可作为提示治疗效果和预测复发可能性的生物指标，例如，外泌体miR-222-3p靶向作用于SOCS3的启动子增加肺癌患者对吉西他滨的耐药及不良预后风险^[57]^。与miR-222-3p作用相反，miR-146a-5p靶向作用于自噬相关蛋白12抑制自噬过程，增加肿瘤细胞对顺铂的敏感性，甚至可逆转A549对化疗的耐药^[83]^。EGFR作为经典突变，获得性耐药是缩短EGFR-TKI敏感患者生存期的原因之一。miR-21诱导Akt磷酸化并激活相应的信号通路，miR-21沉默表达后外泌体介导的Akt活化度降低，同时H827S细胞对吉非替尼的敏感性增加^[84]^，提示miR-21参与EGFR-TKIs的耐药。

**2:** 外泌体miRNA在肺癌中的临床意义 The clinical significance of exosomal miRNA in lung cancer

The author, year	Sample type	Sample size	Extract method	The miRNA species	Clinical significance
Grimolizz F. *et al*, 2017^[65]^	Plasma	45 LC *vs* 31 HC	AUC	miR-126	Down-regulation. Differentiated healthy groups.
Rabinowits G. *et al*, 2009^[66]^	Plasma	27 AD *vs* 9 HC	IMS	miR-17-3p, miR-21, miR-106a, miR-146, miR-155, miR-191, miR-192, miR-203, miR-205, miR-210, miR-212, miR-214	Up-regulation. Differentiated healthy groups.
Kim JE. *et al*, 2018^[67]^	BALF	13 AD *vs* 5 HC	ExoQuick	miR-155, miR-191, miR-192, miR-203, miR-205	Up-regulation. miR-126 and let-7a differentiated healthy groups.
Giallombardo M. *et al*, 2016^[68]^	Plasma	12 NSCLC *vs* 6 HC	AUC	miR-30b, miR-30c, miR-103, miR-195, miR-221, miR-222, miR-122, miR-203	In the lung cancer group, the former 6 miRNAs were up-regulated and the latter 2 were down-regulated, differentiating the healthy group.
Zhou X. *et al*, 2016^[69]^	Plasma	30 LC *vs* 10 HC	ExoQuick	miR-19b-3p, miR-21-5p, miR-221-3p, miR409-3p, miR-425-5p, miR-584-5p	Up-regulation. As a diagnostic marker for lung adenocarcinoma in Asian populations.
Zhang Y. *et al*, 2019[^[70]^	Serum	72 NSCLC *vs* 4 7 HC	ExoQuick	miR-17-5p, miR-18a-5p, miR-19a-3p, miR-19b-1-5p, miR-20a-5p, miR92a-1-5p	Up-regulation. miR-17-5p differentiated healthy groups.
Chen L. *et al*, 2020^[71]^	Serum	65 AD *vs* 65 HC	AUC	MiR-7977	Up-regulation. As a diagnostic marker for adenocarcinoma.
Cazzoli R. *et al*, 2013^[72]^	Plasma	50 AD *vs* 30 LGs *vs* 25 HC	ExoQuick	miR-378a, miR-379, miR-139-5p, miR-200b-5p, miR-151a-5p, miR-30a-3p, miR-200b-5p, miR-629, miR-100, miR-154-3p	Up-regulation. The former 4 distinguish nodules (lung adenocarcinoma and carcinoma) from non-nodules (healthy former smokers): the latter 6 identified lung adenocarcinoma and granuloma.
Hydbringa P. *et al*, 2018^[73]^	PE	18 AD *vs* 10 inflamation	AUC	miR-200	Up-regulation. Distinguish between adenocarcinoma and inflammatory lesions.
Lin J. et al 2016^[74]^	Plasma	9 pneumonia *vs* 9 TB *vs* 9 LC	AUC	27 kinds of miRNAs	Up-regulation. miR-205-5p and miR-200b were in the lung cancer group, and miR-378i was in the pneumonia group.
Jin X. *et al*, 2017 ^[75]^	Plasma	106 LC *vs* 42 HC	AUC	miR-181-5p, miR-30a-3p, miR-30e-3p, miR-361-5p, miR-10b-5p, miR-15b-5p, miR-320b	Up-regulation. The former 4 were specific for adenocarcinoma; the latter 3 were specific for squamous cell carcinoma.
Rodriguez M. *et al*, 2014^[76]^	Plasma, BALF	30 NSCLC *vs* 75 nontumor	AUC	miR-19b-3p, miR-21-5p, miR-409-3p, miR-181-5p, miR-30e-3p, miR-361-5p, miR-19b, miR-30b, miR-151a-5p, miR-100, miR-96, miR-126, miR-19a	Up-regulation. Plasma specificity: miRNA, miR-126 and miR-144; BALF specificity: miR-302 and miR-144.
Wu H. *et al*, 2016^[77]^	Serum	BEAS-2B, A549, PC9, H1299	AUC	miR-96	Upregulated. miR-96 was positively correlated with high-grade and metastatic lung cancer.
Ma YZ. *et al*, 2019^[78]^	Serum	19LC	ExoQuick	miR-425-3p	Upregulated. Platinum chemotherapy has poor efficacy and can be used as a prognostic biomarker.
Liu Q. *et al*, 2017^[79]^	Plasm	10 AD *vs* 10 HC	AUC	miR-23b-3p, miR-10b-5p, miR-21-5p	Upregulated. Associated with poor prognosis.
Xu S. *et al*, 2019^[80]^	Plasma	43 LC *vs* 20 HC	AUC	miR-32	Upregulated. Longer OS and PFS.
He S. *et al*, 2019^[81]^	Serum	A549 BEAS-2B	AUC	miR-499a-5p	Upregulated. Highly metastatic cell lines.
Dejima H. *et al*, 2017^[82]^	Plasma	195 post-operation *vs* 6 recurrence	AUC	miR-21, miR-4257	Upregulated. As a biomarker for predicting recurrence after radical surgery.
AUC: analytical ultracentrifugation; IMS: immunomagnetic bead-based separation; PE: pleural effusion; TB: tuberculosis; BALF: bronchoalveolar lavage fluid; miRNA: microRNA: LG: lung granulomas; AD: Adenocarcinoma; OS: overall survival; PFS: progression-free survival.					

## 5 总结及展望

综上所述，外泌体源性蛋白及miRNA在肺癌发病中发挥重要作用，肺癌患者早期外泌体源性蛋白、miRNA含量及表达谱区别于健康人，且不同病理类型及不同疾病间存在表达谱的差异，因此通过外泌体蛋白及miRNA的检测有望在肺癌的早期诊断和预后评估中实现新的突破。但当前外泌体研究过程中尚存在一些问题：一是外泌体的分类尚无世界范围内统一的标准，不过近年来外泌体的分离、验证及保存方式在逐渐向ISEV认可的标准靠拢；二是外泌体特异性蛋白和miRNA作为诊断标志物的研究日益增多，研究结果重叠的不多，但目前来看针对特定蛋白质或者miRNA的研究增多，提示外泌体研究的广度减小、深度增加。总的来说，外泌体在肺癌相关领域的研究有很大进展空间，仍需进行大量的研究工作促进外泌体研究成果早日转化。
